# Overground walking patterns after chronic incomplete spinal cord injury show distinct response patterns to unloading

**DOI:** 10.1186/s12984-018-0436-1

**Published:** 2018-11-12

**Authors:** Christopher Schmidt Easthope, Luca Renato Traini, Lea Awai, Martina Franz, Georg Rauter, Armin Curt, Marc Bolliger

**Affiliations:** 1 Spinal Cord Injury Center, Balgrist University Hospital, Forchstrasse 340, CH-8008 Zürich, Switzerland; 2 Sobell Department of Motor Neuroscience and Movement Disorders, University College London, London, UK; 3 BIROMED-Lab, Department of Biomedical Engineering, University Basel, Basel, Switzerland

**Keywords:** Body weight support, Gait pattern, Spinal cord injury, Walking, Unloading

## Abstract

**Background:**

Body weight support (BWS) is often provided to incomplete spinal cord injury (iSCI) patients during rehabilitation to enable gait training before full weight-bearing is recovered. Emerging robotic devices enable BWS during overground walking, increasing task-specificity of the locomotor training. However, in contrast to a treadmill setting, there is little information on how unloading is integrated into overground locomotion. We investigated the effect of a transparent multi-directional BWS system on overground walking patterns at different levels of unloading in individuals with chronic iSCI (CiSCI) compared to controls.

**Methods:**

Kinematics of 12 CiSCI were analyzed at six different BWS levels from 0 to 50% body weight unloading during overground walking at 2kmh^− 1^ and compared to speed-matched controls.

**Results:**

In controls, temporal parameters, single joint trajectories, and intralimb coordination responded proportionally to the level of unloading, while spatial parameters remained unaffected. In CiSCI, unloading induced similar changes in temporal parameters. CiSCI, however, did not adapt their intralimb coordination or single joint trajectories to the level of unloading.

**Conclusions:**

The findings revealed that continuous, dynamic unloading during overground walking results in subtle and proportional gait adjustments corresponding to changes in body load. CiSCI demonstrated diminished responses in specific domains of gait, indicating that their altered neural processing impeded the adjustment to environmental constraints. CiSCI retain their movement patterns under overground unloading, indicating that this is a viable locomotor therapy tool that may also offer a potential window on the diminished neural control of intralimb coordination.

## Background

In lower limb rehabilitation of patients with central nervous system (CNS) disorders such as incomplete spinal cord injury (iSCI), gait training with or without body weight support (BWS) is an established intervention [1, 2]. Evidence for recovery induced by locomotor training has been extensively demonstrated in animal models [3, 4]. Underlying this recovery is the neuroplasticity of spinal networks: Repetitive activation of task-specific input-output relationships that result in successful function favor remodeling, leading to neural adaptation in response to the injury [5, 6]. In humans with iSCI, there are numerous published investigations that indicate that gait training leads to accelerated recovery of walking function [7–9]. Compared to the extensive gains observed in animals however, the functional improvements in humans remain limited [10]. This is attributed to various factors, including species-specific anatomy and neurophysiology [11], low sensitivity of outcome measures [12] and suboptimal dosing/timing of the intervention [13]. In coherence with the concept of exploiting the neuroplasticity of residual circuits, it is established that training must be task-specific, active and tailored to the individual patient’s capacity [5].

Recent experiments in rats with iSCI have demonstrated that larger functional gains in basic and skilled locomotion are achieved when training is conducted overground as opposed to training on a treadmill [14]. This implies that the mechanisms driving neural recovery are more actively challenged during overground locomotion training, potentially due to subtle differences on the treadmill including the lack of goal direction and the more passive nature of hip extension (especially during fixed-point unloading) [15, 16]. A case study [17] and more recent review [7] conclude that, in order to be maximally task-specific, neurological locomotor rehabilitation should be optimally conducted overground [5]. While this is readily possible with patients who can support their own body weight, severely affected patients are dependent on BWS. These insights have inspired the development of different ceiling-mounted overground BWS systems [18, 19]. The design of overground BWS systems is challenging however, and the reduction of collateral interaction forces, which potentially impede a patient’s walking pattern and body dynamics leading to distorted afferent and efferent signaling, remains a central point [20]. Low transparency of the BWS system in medio-lateral direction can for instance result in a pendulum-like motion around the point of support during walking [21] influencing the regulation of body sway [22, 23].

For effective rehabilitation, patients should hence train overground walking with a device that offers support and safety while allowing natural motion without perturbing forces in addition to the necessary BWS. In this light, we developed the FLOAT, a cable robot that provides overground BWS with minimal interaction forces to allow unimpeded locomotor training [24, 25]. Briefly, the patient is attached to a central node that is suspended via 4 cables, each deflected over a rail-mounted passive trolley and actuated by its own motor. By tensing all 4 cables, vertical BWS is generated. By tensing the anterior cables more than the posterior, additional forward forces can be applied. Constant and smooth BWS is supplied by sensing the forces acting on each cable at the node and relaying this information to a real-time processor that adapts the individual motor torques. In this way, a constant vertical (and/or forward, and/or sideward) force can be generated. When the node is moved by the patient, the distribution of forces at the sensors changes and the node position is updated to restore equilibrium. This leads to low interaction forces both in the first prototype [24] used in this study and a second, commercialized version [26] (The FLOAT, LME, Switzerland).

Unloading in a treadmill setting has been previously described to induce kinematic modifications including reduced relative stance phase, step length, and hip and knee ranges of motion [27–32]. Experiments investigating kinematics in healthy cohorts walking overground using a cable-propelled gantry system [33–35] and an overhead support system (FLOATv1) [25] have led to similar conclusions. However, it is unknown how unloading during overground walking affects gait patterns in individuals with iSCI. While there is inherent robot-human interaction that can potentially distort the gait pattern in a similar manner to controls, unloading also intrinsically engenders reduced cutaneous and loading afferents through the reduced contact forces [36]. Loading afferents are one of the main drivers for spinal networks that have long been considered especially important for chronic iSCI gait [37–39]. A reduction of the loading signal may therefore result in a degradation of the stereotyped gait pattern that is less dependent on neural circuits rostral to the lesion site [40–42]. If overground unloading is to be a useful tool in gait rehabilitation for those patients that retain volitional movement capacity but lack the strength to support their own body weight, it must be ensured that the gait pattern employed during unloading, i.e. with reduced loading information, remains physiological.

This study aimed at describing the kinematic changes induced by different levels of unloading during overground locomotion in an iSCI population. Specifically, we were interested if controlled, continuous, dynamic unloading would lead to changes in individuals with chronic iSCI (CiSCI) walking patterns and how these changes would compare to those previously reported from an able-bodied control group [25]. It was hypothesized that CiSCI show greater changes in their walking patterns in response to body unloading compared to those reported in controls.

## Methods

Fifteen CiSCI who were community ambulators were recruited and gave written informed consent prior to participating in this study. Inclusion criteria were an iSCI at least 3 months prior to inclusion and the ability to walk 10 m without or with only minor (e.g. stick) walking aids in under 20 s. CiSCI were excluded if they had any secondary neurological diagnosis, chronic pain, skin inflammation, or orthopedic or cardiovascular diagnosis or if they were still in active therapy. Control data was extracted from a previously established database of control subjects [25]. The study was approved by the local ethics committee of the Canton of Zurich (KEK Nr. PB_2016–00228) and was conducted in accordance with the Declaration of Helsinki.

To provide BWS, an in-house developed cable robot was employed [24]. The continuous, dynamic, vertical BWS was scaled to each subject’s body weight. Gait kinematics were recorded using a passive infrared optical motion capture system (T-series, Vicon, UK), sampling at 200 Hz.

The CiSCI underwent a physical examination by a physician to determine their functional status prior to enrolling into the study. This encompassed walking measures in form of a ten-meter walking test (10mWT) to assess maximal walking speed, functional measures in terms of motor (lower extremity motor scores [LEMS]) and sensory scores (American Spinal Injury Association [ASIA] Impairment Scale [AIS]), and proprioceptive testing. Proprioception tests included assessment of vibration sense using a graded Rydel-Seiffer tuning fork at the Hallux dorsi, the Malleolus and the Patella on both sides and scoring the patient on a scale of 0 to 8 [43]. Furthermore, position sense of the Hallux was recorded on a scale of 0 to 2 [44] and a Romberg test [45] was performed. Information about the latencies of tibial nerve sensory evoked potentials (SEPs) and latencies of motor evoked potentials of the tibialis anterior (MEPs) were retrieved from current clinical records to improve patient characterization.

Prior to the experimental conditions, all participants were weighed and characteristic anatomical measures retrieved to enable accurate calculation of joint center positions [46, 47]. They then were equipped with a BWS harness that was modified to not obscure the pelvis landmarks and a passive reflective marker set was attached to these and other bony landmarks (Plug-in-Gait v3.0, Vicon, UK). For the remainder of the experiment, participants were attached to the BWS-system for unloading.

Participants were required to walk 20 steps in multiple passes of the 2 × 8 m workspace at six different clinically relevant BWS levels in random order (baseline (0%); 10%; 20%; 30%; 40%; 50% BWS). At the baseline level (0%), a small unloading force (~ 4 kg) was applied to enable fluid tracking of the node. Walking speed was acoustically indicated at 0.56 m/s (2 km/h) with a tolerance of 0.14 m/s (0.5 km/h) as this represented the minimal inclusion speed for the CiSCI group: A low tone was given when participants were walking too fast and a high tone when they were too slow. The speed range was employed especially so that CiSCI could walk through the workspace without being constantly corrected due to within-stride fluctuations of node velocity. For each new BWS level, familiarization passes were conducted until speed variability declined to the point that no acoustic warnings were triggered during a pass (no more than 2 passes per BWS level were necessary for any patient).

### Analysis

Marker position data were filtered using Woltring’s quintic spline [48] with a laboratory-specific squared mean standard error of 15mm^2^. Data were then synthesized with anatomical measurements to define segments and joint center locations enabling the calculation of joint angles using a clinical biomechanical model (Plug-in-Gait 3.0, Vicon, UK). Heel strike and toe off events were set manually by a single analyst using heel and toe marker trajectories and velocities. Subsequently the angle and position data were segmented into steps (heel strike to ipsilateral heel strike), time-normalized via linear interpolation to 500 points, and characteristic step parameters were extracted using custom Matlab routines (2016b, the Mathworks, USA). Step parameters included: speed, step length, step width, relative double support phase, step time, and joint (hip, knee, and ankle) ranges of motion. Ranges of motion of the center of mass (CoM) and of trunk inclination were extracted in antero-posterior (AP) and medio-lateral (ML) direction to investigate effects of the BWS system and harness on walking posture. To enable the comparison of waveforms independently of shifts in relative stance phase, joint motion patterns and intralimb coordination were separately analyzed using a stance-swing linear normalization. Joint motion patterns were averaged over all steps in one condition into a characteristic waveform and then compared on the group level. Intralimb coordination was depicted as correspondence between two joint angles in form of cyclograms (hip-knee, knee-ankle). Differences in cyclogram shape between groups and conditions were described by calculating the square root of the sum of squared distances (SSD) from the mean cyclogram at baseline BWS in the control group. Intra-subject consistency in intralimb coordination was quantified using the angular component of coefficient of correspondence (ACC). These approaches have previously been successfully used to sensitively and reliably discern changes in locomotor behavior of iSCI populations [49–51].

CiSCI data was assigned to more and less affected sides based upon a summation of sensory and motor scores for each of the lower limbs. Laterality, however, was mainly determined by the sensory component, as the motor scores were in all cases almost symmetrical. Reference data from healthy controls was averaged for both sides. All side-dependent results are reported for the more affected limb unless the limbs showed strikingly different patterns.

### Statistical analysis

All statistics for time-discrete parameters were calculated in SPSS (v24, IBM Corp., USA). Age, height and weight were normality-verified and compared between groups with unpaired t-tests. All other parameters were verified for normality (Shapiro-Wilk), homogeneity of variances (Levene’s), and sphericity (Mauchly), and subsequently compared between BWS levels using a two-way ANCOVA (group x BWS) with one repeated factor (BWS) and age as a covariate. If a significant interaction or BWS main effect was detected, the factor group was fixed and the model repeated. In all cases, simple a-priori contrasts to baseline with a Sidak correction were performed and are reported when eligible. Continuous data was compared in Matlab analogously using a two-way ANOVA (group x BWS) with one repeated factor (BWS) from the spm1d package (v0.4.0; www.spm1d.org) for one-dimensional (time-continuous) data [52, 53]. Main group BWS effects were investigated via paired t-tests with appropriate Bonferroni correction (*p* = 0.05/5) within each group. All results are reported as means ± standard deviation (SD).

## Results

### Population

From the recruited population, 12 CiSCI (age: 51 ± 14 yrs., height: 176 ± 7 cm, weight: 78 ± 17 kg, 3 females) completed the full protocol and were included in the analysis (Table 1). The three excluded CiSCI could not manage the baseline condition of walking 20 strides at 0.56 ms^− 1^ without unloading within the tolerance range. Previously published control data [25] of 18 subjects (age: 29 ± 5 yrs., height: 174 ± 9 cm, weight: 71 ± 12 kg, 9 females) was used as a comparator for CiSCI responses to unloading. While age was significantly different between groups (t(1,28) = 6.35; *p* = 0.000), height (t(1,28) = 0.69; *p* = 0.458) and weight (t(1,28) = 1.32; *p* = 0.458) were similar. In comparison to clinical reference values [54], tibial SEP (46 ± 5 ms (CiSCI); 41 ± 3 ms (clinical reference data for mean group height)) and MEP (32 ± 3 ms (CiSCI); 28 ± 2 ms (clinical reference data for mean group height)) latencies were slightly increased in CiSCI. Specifically, in mean, CiSCI SEP latencies were 1.75 ± 1.5 SDs and patient MEP latencies were 2.1 ± 1.9 SDs above their height matched reference values. Walking speed ranged from 0.42–0.67 m/s in CiSCI (55 ± 22% of individual maximal velocity) and 0.5–0.62 m/s in the control group (23 ± 7% of reported maximal velocity [55]). Unloading induced a simple main effect on walking speed (F(5,130) = 11.110, *p* = 0.000) in the presence of a group x BWS interaction effect (F(5,130) = 6.529, *p* = 0.000). Both groups reduced their speed with increasing unloading (F(5,50) = 5.867, *p* = 0.000 (CiSCI); F(5,85) = 3.431, *p* = 0.007 (controls)), however this affected CiSCI more strongly than controls (Table 2).

**Table 1:** CiSCI characteristics

	Characteristics							ASIA			Sensory			SEP		MEP	
#	Sex	Age	Lesion Level	TSI [yrs]	Height [cm]	Weight [kg]	10mWT [s]	LEMS (50)	LT (56)	PP (56)	POS (4)	VIB (48)	RB (2)	L [ms]	R [ms]	L [ms]	R [ms]
01	m	35	C2	1.16	177	74.7	6.97	49	34	42	4	40	0	45	45	32	31
02	m	68	C4	0.5	184	99.4	8.91	47	56	52	3	12	1	42	41	29	29
03	m	64	C2	11	172	88.8	9.15	48	40	44	4	12	2	52	52	43	41
04	m	29	C6	1.25	176	63.7	17.21	42	40	33	4	28	2	41	43	30	31
05	f	64	C6	4.3	166	48.5	9.27	46	55	52	3	46	1	46	46	34	35
06	m	44	C2	13	188	96.8	10.17	47	11	10	3	12	2	55	52	32	33
07	f	46	T8	0.3	171	94.8	5.78	47	32	20	4	48	0	41	41	29	29
08	f	52	T9	7.25	177	81.1	13.09	40	52	52	3	28	2	44	47	32	31
09	m	68	C7	0.4	167	58.2	6.47	49	56	56	4	42	0	45	42	NA	NA
10	m	38	C6	8.25	182	71.7	7.31	45	30	13	4	34	0	56	54	32	33
11	m	48	C2	13.2	177	77	6.82	48	56	56	4	38	1	43	42	29	29
12	m	63	T9	6.1	17	80	17.41	43	35	15	3	3	2	53	47	35	32

*Characteristics of the individuals with iSCI included in the analysis, especially the time since injury (TSI) and the ten-meter walking test (10mWT) as a measure of walking function. This also includes the summation of the left and right lower extremity motor scores (LEMS), light touch scores (LT), and pin prick scores (PP) along with position sense (POS), vibration sense (VIB) and Romberg (RB) scores. Latencies from sensory evoked potentials (SEP) and cortical motor evoked potentials (MEP) are reported for the tibial nerve (NA: Not assessed). Where appropriate, units and maximal possible scores are indicated in parenthesis*

**Table 2:** *Gait Parameters*

	Parameter	Group	0% BW	10% BW	20% BW	30% BW	40% BW	50% BW	Group BWS effect	Simple BWS effect	Group x BWS
Spatio-temporal	Step Length [mm]	Control	435 (36)	438 (43)	443 (46)	**456 (48)**	444 (46)	**462 (51)**	F(5,85) = 7.477, *p* = 0.000	F(5,130) = 3.066, *p* = 0.012	F(5,130) = 3.116, *p* = 0.011
		CiSCI	418 (85)	431 (76)	423 (69)	421 (73)	416 (79)	420 (81)	F(5,50) = 0.916, *p* = 0.478		
	Step Width [mm]	Control	63 (27)	**59 (25)**	**57 (24)**	60 (27)	66 (30)	70 (31)	F(5,85) = 3.053, *p* = 0.014	F(5,130) = 1.756, *p* = 0.126	F(5,130) = 2.435, *p* = 0.038
		CiSCI	100 (28)	91 (25)	93 (31)	84 (25)	88 (28)	84 (31)	F(5,50) = 1.491, *p* = 0.209		
	Step Time [s]	Control	0.75 (0.06)	0.75 (0.07)	**0.76 (0.08)**	**0.78 (0.09)**	**0.79 (0.09)**	**0.82 (0.10)**	F(5,85) = 11.342, *p* = 0.000	F(5,130) = 6.514, *p* = 0.000	F(5,130) = 0.479, *p* = 0.778
		CiSCI	0.68 (0.09)	0.71 (0.13)	0.69 (0.11)	0.74 (0.10)	0.78 (0.13)	0.79 (0.12)	F(5,50) = 1.453, *p* = 0.222		
	Double Support Phase [%]	Control	0.29 (0.02)	0.29 (0.02)	**0.27 (0.03)**	**0.25 (0.03)**	**0.23 (0.03)**	**0.2 (0.05)**	F(5,85) = 49.294, *p* = 0.000	F(5,130) = 36.528, *p* = 0.000	F(5,130) = 1.354, *p* = 0.245
		CiSCI	0.33 (0.05)	0.33 (0.05)	0.3 (0.04)	0.3 (0.04)	**0.28 (0.06)**	**0.26 (0.05)**	F(5,50) = 6.805, p = 0.000		
	Speed [km/h]	Control	2 (0.07)	2 (0.1)	2 (0.1)	2 (0.1)	2 (0.1)	2 (0.1)	F(5,85) = 3.431, *p* = 0.007	F(5,130) = 11.110, *p* = 0.000	F(5,130) = 6.529, *p* = 0.000
		CiSCI	2.1 (0.2)	2.1 (0.2)	2.1 (0.2)	2 (0.2)	1.9 (0.2)	**1.9 (0.2)**	F(5,50) = 5.867, *p* = 0.000		
Posture	A-P Sway [°]	Control	23 (5)	22 (6)	**19 (4)**	**18 (4)**	**16 (4)**	**17 (4)**	F(5,85) = 16.366, *p* = 0.000	F(5,130) = 5.353, *p* = 0.000	F(5,130) = 2.877, *p* = 0.017
		CiSCI	30 (14)	29 (14)	28 (12)	25 (8)	26 (8)	24 (9)	F(5,50) = 0.637, *p* = 0.673		
	CoM AP motion [mm]	Control	860 (70)	870 (83)	880 (88)	**910 (95)**	890 (90)	**920 (98)**	F(5,85) = 6.561, *p* = 0.000	F(5,130) = 1.786, *p* = 0.119	F(5,130) = 4.911, *p* = 0.000
		CiSCI	820 (130)	840 (120)	830 (120)	840 (120)	820 (150)	820 (150)	F(5,50) = 1.471, *p* = 0.216		
	M-L Sway [°]	Control	20 (6)	20 (6)	22 (6)	**23 (7)**	**24 (5)**	**24 (7)**	F(5,85) = 4.768, *p* = 0.001	F(5,130) = 5.333, *p* = 0.000	F(5,130) = 1.066, *p* = 0.382
		CiSCI	27 (11)	26 (11)	30 (12)	27 (9)	30 (11)	29 (12)	F(5,50) = 2.173, *p* = 0.072		
	CoM ML motion [mm]	Control	43 (9)	**40 (10)**	**39 (10)**	43 (15)	**37 (9)**	**38 (13)**	F(5,85) = 2.760, *p* = 0.023	F(5,130) = 7.016, *p* = 0.000	F(5,130) = 1.568, *p* = 0.173
		CiSCI	57 (12)	52 (15)	**49 (12)**	49 (16)	**44 (13)**	**41 (13)**	F(5,50) = 4.601, *p* = 0.002		
Single Joint	Hip ROM [°]	Control	32 (3)	33 (3)	32 (3)	32 (4)	**30 (3)**	**30 (4)**	F(5,85) = 6.397, *p* = 0.000	F(5,130) = 3.794, *p* = 0.003	F(5,130) = 0.684, *p* = 0.636
		CiSCI	34 (6.6)	34 (6.8)	32 (6.4)	34 (7.6)	33 (7.4)	31 (7.2)	F(5,50) = 0.901, *p* = 0.488		
	Knee ROM [°]	Control	47 (5)	47 (5)	**46 (4)**	47 (5)	**46 (5)**	46 (5)	F(5,85) = 3.113, *p* = 0.013	F(5,130) = 8.920, *p* = 0.000	F(5,130) = 1.168, *p* = 0.328
		CiSCI	50 (9)	50 (9)	49 (8)	48 (10)	47 (9)	**45 (10)**	F(5,50) = 5.355, *p* = 0.001		
	Ankle ROM [°]	Control	22 (4)	22 (4)	22 (4)	23 (4)	23 (7)	**26 (7)**	F(5,85) = 6.020, *p* = 0.000	F(5,130) = 3.774, *p* = 0.000	F(5,130) = 1.328, *p* = 0.256
		CiSCI	20 (5)	21 (5)	20 (4)	24 (10)	24 (12)	24 (11)	F(5,50) = 0.853, *p* = 0.519		
Intralimb	SSD Hip-Knee [A.U.]	Control	4.9 (1.9)	4.9 (1.4)	5.4 (1.9)	**6.2 (1.7)**	**8.4 (3.1)**	**12 (3.5)**	F(5,85) = 43.405, *p* = 0.000	F(5,130) = 20.440, *p* = 0.000	F(5,130) = 8.479, *p* = 0.000
		CiSCI	8.3 (3.6)	8.7 (3.9)	7.8 (3.5)	8.7 (3.2)	9.4 (5.1)	9.8 (4.9)	F(5,50) = 1.255, *p* = 0.298		
	SSD Knee-Ankle [A.U.]	Control	4.5 (1.5)	4.6 (1.3)	5.4 (2)	**6.5 (2.1)**	**8.9 (3.8)**	**12 (4.2)**	F(5,85) = 31.107, *p* = 0.000	F(5,130) = 16.615, *p* = 0.000	F(5,130) = 7.203, *p* = 0.000
		CiSCI	10 (4.9)	11 (4.9)	7.7 (2.7)	10 (4.5)	11 (5.2)	11 (4.9)	F(5,50) = 2.374, *p* = 0.052		
	ACC Hip-Knee [A.U.]	Control	0.97 (0.01)	0.97 (0.01)	0.97 (0.01)	0.97 (0.01)	**0.96 (0.02)**	**0.95 (0.02)**	F(5,85) = 8.685, *p* = 0.000	F(5,130) = 6.326, *p* = 0.000	F(5,130) = 0.292, *p* = 0.917
		CiSCI	0.96 (0.03)	0.96 (0.03)	0.95 (0.03)	0.95 (0.04)	0.95 (0.03)	0.94 (0.03)	F(5,50) = 1.263, *p* = 0.295		
	ACC Knee-Ankle [A.U.]	Control	0.95 (0.01)	0.95 (0.02)	**0.94 (0.02)**	**0.94 (0.02)**	**0.92 (0.03)**	**0.9 (0.04)**	F(5,85) = 14.584, *p* = 0.000	F(5,130) = 18.104, *p* = 0.000	F(5,130) = 0.345, *p* = 0.885
		CiSCI	0.93 (0.04)	0.93 (0.04)	0.91 (0.04)	0.91 (0.06)	**0.88 (0.05)**	**0.87 (0.06)**	F(5,50) = 5.742, *p* = 0.000		

*Mean gait parameter values (mean(SD)) in control and CiSCI cohorts at different BWS levels. F-statistics for simple BWS effect and group x BWS interaction are reported from the 2-way ANCOVA with one repeated factor and age as a covariate. The group-split F-statistics are reported as group effects and in case a significant effect was identified,* a-priori *simple contrasts to group baseline (0% BW) with a Sidak correction are indicated through* ***bold*** *formatting (p < 0.05). For CiSCIs, when parameters are side-dependent, only the result of the more affected side is reported unless results diverged significantly between more and less affected side*

### Spatio-temporal parameters

CiSCI adapted their temporal parameters, namely step time and double support phase to increasing BWS similarly to controls (Table 2). A simple main effect of BWS was present in step time (F(5,130) = 6.514, *p* = 0.000) and relative double support phase (F(5,130) = 36.528, *p* = 0.000), however there was no interaction effect between group and BWS (F(5,130) = 0.479, *p* = 0.778 and F(5,130) = 1.354, *p* = 0.245 respectively) (Table 2). Step length showed a simple main effect of BWS (F(5,130) = 3.066, *p* = 0.012) and an interaction effect (F(5,130) = 3.116, *p* = 0.011), with controls increasing their step length (F(5,85) = 7.477, *p* = 0.000) while CiSCI did not (F(5,50) = 0.916, *p* = 0.478). Step width on the other hand did not show a simple main effect of BWS (F(5,130) = 1.756, *p* = 0.126), however a group x BWS interaction was present (F(5,130) = 2.435, *p* = 0.038). Controls developed a local minimal at 10–20%BWS (F(5,85) = 3.053, *p* = 0.014), while CiSCI step width remained unchanged (F(5,50) = 1.491, *p* = 0.209).

### Walking posture

Walking posture was assessed using CoM motion and trunk sway in AP and ML directions (Table 2). In AP, trunk sway showed a simple main BWS effect (F(5,130) = 5.353, *p* = 0.000) and an interaction effect (F(5,130) = 2.877, *p* = 0.017). Controls reduced AP trunk sway (F(5,85) = 16.366, *p* = 0.000), while this was not detected in CiSCI (F(5,50) = 0.637, *p* = 0.673). AP CoM motion did not show a simple main BWS effect (F(5,130) = 1.786, *p* = 0.119), however an interaction was present (F(5,130) = 4.911, *p* = 0.000). Controls increased AP Com (F(5,85) = 6.561, *p* = 0.000), while this remained unchanged in CiSCI(F(5,50) = 1.471, *p* = 0.216). In medio-lateral direction, a simple main effect on trunk sway (F(5,130) = 5.333, *p* = 0.000) and CoM motion (F(5,130) = 7.016, *p* = 0.000) was detected, however there was no significant interaction effect (F(5,130) = 1.066, *p* = 0.382 and F(5,130) = 1.568, *p* = 0.173 respectively).

### Ranges of motion

**Fig. 1:**
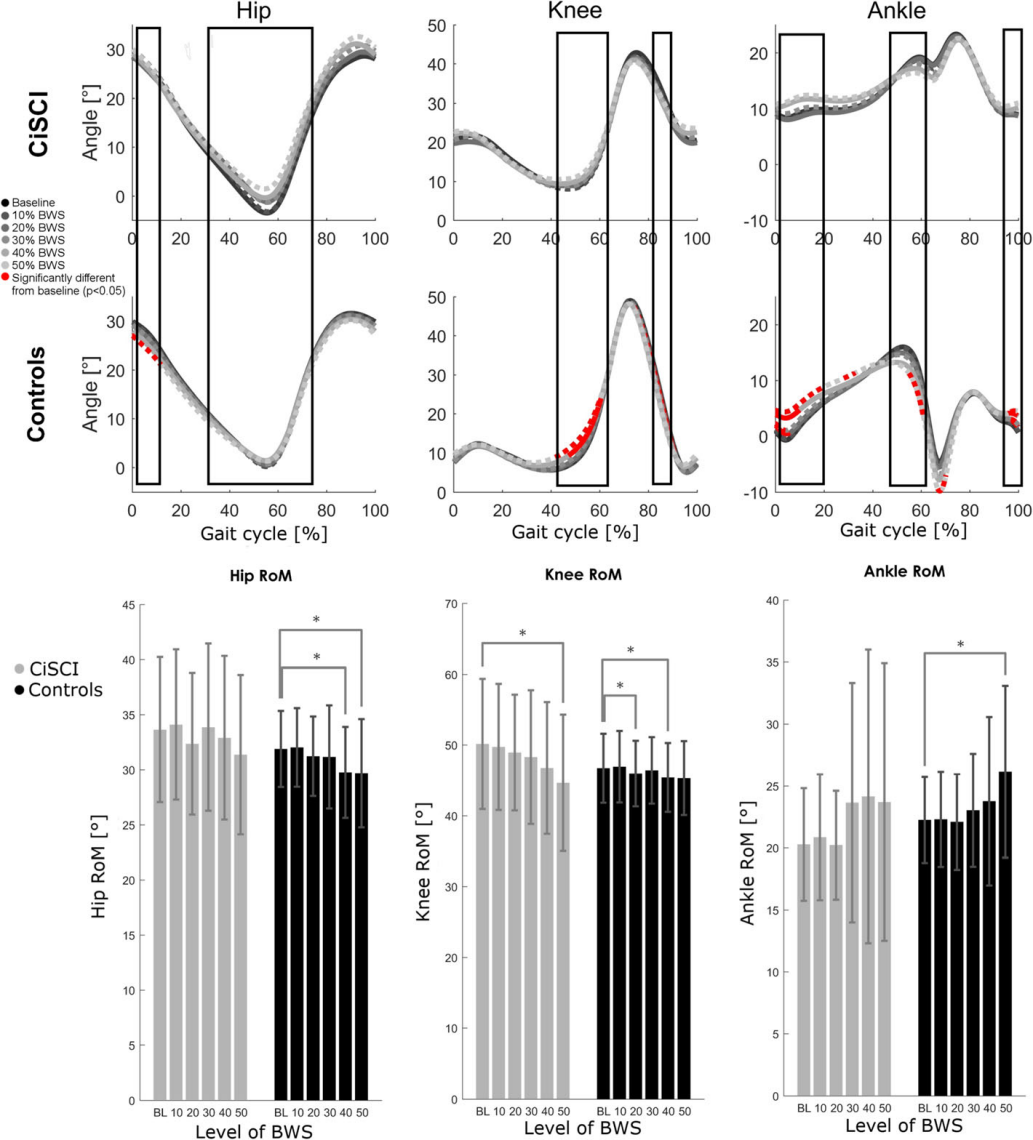
Lower limb angular waveforms & ranges of motion. Angular waveforms of hip, knee and ankle joints in the sagittal plane in controls (middle) and iSCI patients (top) at 10%, 30% and 50% BWS with increasing shades of gray. Simple main effects of BWS are indicated by the boxed areas for each joint. Significant deviation from the baseline condition within each group is indicated with red coloring for the affected section of the gait cycle. Range of motion (bottom) is depicted for hip, knee and ankle joints (sagittal plane) for patient (gray) and control (black) populations at all levels of BWS. Bars indicate group means with 1SD. Significance is detailed as *: *p* < 0.05

Ranges of motion (ROMs) of lower extremity joints in the sagittal plane responded similarly to unloading (Table 2, Fig. 1). A simple main BWS effect was detected for hip (F(5,130) = 3.794, *p* = 0.003), knee (F(5,130) = 8.920, *p* = 0.000) and ankle (F(5,130) = 3.774, *p* = 0.000) joints, however there were no interaction effects (F(5,130) = 0.684, *p* = 0.636; F(5,130) = 1.168, *p* = 0.328; F(5,130) = 1.328, *p* = 0.256 respectively).

### Joint motion patterns

Unloading induced simple main effects in joint motion patterns of the hip (F* = 3.152, *p* < 0.044), knee (F* = 3.152, *p* < 0.043) and ankle (F* = 3.567, *p* < 0.025). At the knee these effects were centered around the toe-off event, while in the ankle they were mainly during the stance phase and in early swing (Fig. 1). Significant interaction effects in the joint waveforms were present at the hip level (F* = 3.152, *p* < 0.039), and the ankle level (F* = 3.567, *p* < 0.012). but not at the knee (F* = 3.152, *p* > 0.05). The interaction effect for the hip joint was strongest during the swing phase and in early stance, while the ankle interaction was most prominent in late stance.

### Cyclograms

Intralimb coordination quantified via SSD showed a simple main BWS effect for both hip-knee (F(5,130) = 20.440, *p* = 0.000) and knee-ankle (F(5,130) = 7.203, *p* = 0.000) couplets. An interaction effect was present in both parameters (F(5,130) = 8.479, *p* = 0.000 and F(5,130) = 7.203, *p* = 0.000 respectively). While SSD increased with increasing unloading in controls (F(5,85) = 43.405, *p* = 0.000; F(5,85) = 31.107, *p* = 0.000), no patient response was detected (F(5,50) = 1.255, *p* = 0.298; F(5,50) = 2.374, *p* = 0.052). Shape consistency determined via ACC demonstrated a simple main BWS effect for hip-knee (F(5,130) = 6.326, *p* = 0.000) and knee-ankle (F(5,130) = 18.104, *p* = 0.000) couplets, however no interaction effects were detected (F(5,130) = 0.292, *p* = 0.917 and F(5,130) = 0.345, *p* = 0.885 respectively).

## Discussion

Application of BWS constitutes a promising approach in the rehabilitation of walking in patients suffering from CNS disorders such as iSCI or stroke. Within the focal group of patients that have voluntary control over their lower limbs, however lack the ability to ambulate freely, the FLOAT can provide transparent BWS with minimal interaction forces. Applying up to 50% BWS to CiSCI during overground walking resulted in subtle changes of walking kinematics that are largely comparable to those observed in healthy controls. While these BWS-induced changes were evident in spatio-temporal parameters such as step length and relative stance phase duration, joint motion patterns and intralimb coordination were modulated to a lower degree in iSCI than in controls. These results are contrary to our initial expectations that BWS would have a larger effect on the walking kinematics of CiSCI compared to controls. This indicates that using overground BWS for up to 20% BW unloading has no discernable effect on walking patterns and even high unloadings of 50% BW do not fundamentally distort walking kinematics. Furthermore, the differences in modulation of intra-limb coordination may provide an opportunity to specifically target this domain in iSCI rehabilitation.

### Spatio-temporal adaptation

As one of the salient characteristics of gait, the adjustment of step length and step time are prerequisites in handling changes in walking conditions. With increased unloading, step time is progressively increased in both controls and iSCI subjects. Similar observations from the literature have not resulted in a conclusive explanation overground [33, 56] or on the treadmill [27, 29–31, 57]. One possible explanation hinges on the dynamic similarity framework, assuming that the Froude number, an anatomy-independent measure of velocity dependent on leg length and gravitation, has an optimal value of around 0.25 for walking [57, 58].

$$ Fr=\frac{\frac{m^{\ast }{v}^2}{h}}{m^{\ast}\mathsf{g}}=\frac{v^2}{{\mathsf{g}}^{\ast }h}=\frac{{\left({sl}^{\ast } sf\right)}^2}{{\mathsf{g}}^{\ast }h} $$

*Fr: Froude number, m: mass[kg], v: velocity[ms* ^*− 1*^ *], h: leg length[m], g: gravitational constant[ms* ^*− 2*^ *], sl: step length[m], sf: step frequency[Hz].*

Reduced “gravity” through unloading while maintaining walking velocity should not disturb the relationship between step length and stride frequency. Applying unloading at the trunk, however, results in an interesting interaction: gravity acting on the swing leg remains at a normal level (9.81 ms^− 2^), while stance phase dynamics are subjected to reduced gravity plus potential robot interaction forces [59]. This typically results in a small but robust [57] reduction of the duty factor, driven mainly through an increase of step time [27, 29–31, 33, 56] while maintaining stance time. To maintain a given speed, relative stride length must be increased, disturbing the developmentally stable relationships between stride length, frequency and walking speed [60]. This behavior is adopted by control subjects. CiSCI on the other hand, display the reduced duty cycle, however they do not modulate step length as appropriately as controls, also leading to the slight reduction in walking speed registered at 50% BWS (Table 2).

Changes in step width were inconclusive in both controls and CiSCI; although an interaction between BWS and group was detected, there was no simple main effect of unloading. This could be due to interaction with the robot in medio-lateral direction, alluding to the complex interaction between BWS, lateral stability, and robot interaction forces [21, 23]. Transparency analysis leads us to somewhat discount the pendulum effect of overhead suspension [26], however the interplay between vertical unloading and step width and other parameters associated with walking stability merits further investigation in healthy subjects. CiSCI retained a large variability in step width throughout all unloading conditions, potentially masking any meaningful responses to unloading. Mean step width trended toward reduction from a pathologically high baseline, hinting that unloading enables CiSCI to walk with a narrower base of support.

### Walking posture effects

We quantified CoM displacement and trunk sway to understand how transparent BWS affects posture during walking and if this is different between CiSCI and controls. The simple main effect of BWS on ML CoM motion and AP and ML trunk sway indicates that the BWS system has some effect on walking posture and trunk control. As the unloading increases, trunk sway increases in both planes while ML motion of the CoM is reduced, and AP in controls remains unchanged. CiSCI adopt a different response pattern in both AP parameters, perhaps due to the shorter initial step length which would require less momentum transfer from the trunk to the pelvis to induce the weight shift. CiSCI also demonstrated a greater AP trunk sway in the initial condition, perhaps compensating for changes in the peak force generation of the trunk muscles. Furthermore, walking speed was reduced to a slightly greater degree in CiSCI under unloading. In summary, CiSCI may be more susceptible to the dynamics of the robot and the constraints of the harness – especially in directions where higher accelerations are necessary [25]. The overhead design and harness location potentially forced a more upright posture and allowed less forward transfer of weight to the leading foot.

The changes in medio-lateral parameters with increased unloading were reflected in both groups. This contrasted with step width, which demonstrated a local minimum at 20%BWS for controls and progressive, but non-significant reduction in CiSCI. This indicates that both groups increasingly opt to retain their CoM closer to the medial edge of their base of support with increasing BWS, alluding to a complex interaction between unloading and frontal plane dynamics [61]. Also, the superior-inferior motion speed of the BWS robot further complexifies the interaction, as the tension is not perfectly equal at all time points. This leads to subtle changes in support levels and momentum transfers in different phases of gait that likely influence the spatio-temporal structure of walking stability, however these interactions are challenging to accurately quantify.

### Joint motion patterns

With increasing unloading, we observed increasing temporal shifts of the angle-time traces in both CiSCI and controls. To adequately compare joint motion patterns, stance and swing phase were normalized and interpolated separately to remove temporal effects while preserving time-rank and amplitude information. This enabled the detection of differences in trajectory shape induced through unloading. While the knee joints showed a simple main unloading effect, especially around the toe-off event and during stance, there was no interaction effect. This indicates that in the investigated sample we could not detect different knee strategies between CiSCI and controls in response to the unloading. At the hip joint, unloading induced a simple main effect centered around the toe-off event and there was a strong interaction effect especially during swing and following heel strike. This interaction effect may be driven by the shorter step length of the CiSCI coupled with the changes in walking posture [62]. At the ankle joint, an interaction effect was present especially in late stance where controls emphasized their push off motion while CiSCI showed no adaptation. Unloading CiSCI in the BWS system induced no detectable change in joint motion patterns, while controls exhibited subtle adaptations especially in the ankle joint at high unloading levels. In controls, the changes in ankle joint motion could be interpreted as a task-specific adaptation, necessary to maintain a given speed while optimizing other, salient gait determinants [58, 63, 64]. That we did not detect these adaptations in our cohort of primarily sensory-affected CiSCI could be linked to changes in the integration of load information into the efferent command structure [65, 66]. Models of spinal network activity during gait indicate that load- and shear-sensitive mechanoreceptors on the foot sole along with Golgi tendon organs and muscle spindle afferents contribute significantly to the successful regulation of gait [39, 66]. However, following iSCI, afferent information processing is altered on many levels [67, 68] and is partially replaced with surrogate, redundant information stemming from non-impacted sources [69] such as visual control [70, 71].

### Intralimb coordination

**Fig. 2:**
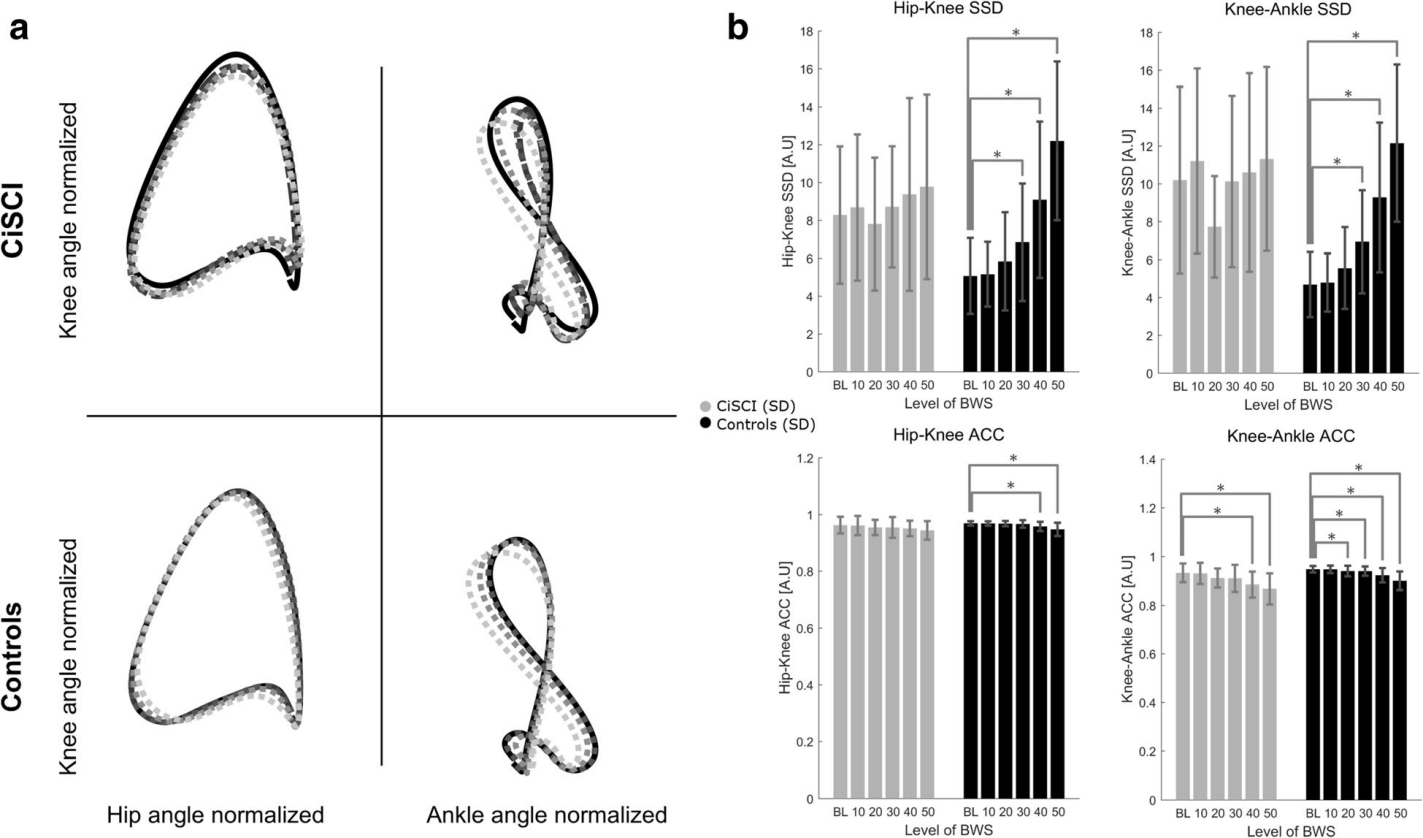
Intralimb coordination in controls and patients under BWS. **a** Response of intralimb coordination patterns to unloading in patients and controls. As a reference, mean baseline control data is shown as a continuous black line. Patient and control mean responses are depicted as dotted lines at 10%, 30% and 50% BWS in increasing shades of gray. Mean patient baseline data is shown as a dashed line. **b** Quantification metrics of the intralimb coordination. Unloading results in a change in SSD in controls (black) for both hip-knee and knee-ankle joint couplets. This coordination pattern remains unaffected in patients (gray) in both couplets. Shape consistency (ACC) is increasingly degraded in both groups with rising unloading. Bars indicate group means with 1SD. Significance is detailed as *: *p* < 0.05

Intralimb coordination patterns have been repeatedly reported to be sensitive readouts of locomotor control in iSCI subjects [49–51]. Knowledge from upper limb [72] and lower limb [73] experiments in humans leads us to interpret multi-joint coordination as a product of proprioceptive integration in the spinal cord at different levels combined with supraspinal efferent drive [74]. Intralimb coordination patterns can be quantified as the form difference from a reference shape (SSD; a form of procrustean shape analysis) [49]. Variability of these patterns can be captured by the angular component of the coefficient of correspondence (ACC; a specialized form of vector coding [51]), which describes the mean dispersion of all sequential point pairs in the cycle. Increased variability in the movement coupling of segments is detected as a decrease in the ACC (range: 0–1). Increased movement variability, especially concerning the linkage of adjacent intralimb segments, can be interpreted as increased neural noise in the generation of synergistic muscle activation patterns. Under unloading, control subjects showed progressive changes in intralimb coupling through increased shape difference and variability (Table 2 and Fig. 2) in both couplets (hip-knee and knee-ankle). In CiSCI, however, SSD remained unchanged in both proximal and distal couplets. No interaction effect was detected for ACC of both couplets, however unloading resulted in a higher coupling variability in both groups. Patient’s lack of modulation of intralimb coupling under unloading may provide access to investigating and challenging this aspect after iSCI.

In synthesis, CiSCI and controls demonstrated similar responses to unloading in terms of spatio-temporal and walking posture parameters. CiSCI, however, retained their baseline intralimb coordination while controls modified this in response to unloading, optimizing their joint coupling patterns to adapt to the unloading while maintaining a given speed. It seems that in a healthy CNS, intralimb patterns are mediated not just at a spinal level, but also integrate a strong influence of supraspinal centers [75]. Once altered following CNS injury however, the coupling of segments has proven remarkably resilient to change. In iSCI patients, for instance, Awai and Curt [12] reported unchanged intralimb walking pattern shapes in iSCI patients throughout their rehabilitation, despite increases in walking speed and reductions in intralimb variability. Similarly, Tepavac and Field-Fote [51] reported improved consistency, yet no systematic change of shape in intralimb coupling following peroneal stimulation coupled with training in 14 iSCI patients. Analogous observations have been made in stroke survivors with lower limb impairment [76–78].

These observations could be driven by alteration of efferent drive induced by changes in supraspinal processing, including the partial loss and replacement of afferent signals. Alternatively, the coupling of segments may be mainly encoded by rhythmic spinal networks and therewith be difficult to modulate via efferent drive, especially when this drive is impaired through a lesion. Both of these two models indicate that when optimizing gait phenotype, especially intralimb coordination, to environmental constraints or when encountering novel tasks, the altered processing present in CiSCI cannot fully compensate for the degraded afferent information. Within this perspective, the response profile of CiSCI to unloading; adapted spatio-temporal parameters, unchanged joint motion patterns and interlimb coordination, may allude to the hierarchy of control in locomotion [79, 80]. Here, neural resources are reserved for task-critical parameters such as spatio-temporal organization of gait and balance control while less critical parameters such as intralimb coordination are preempted from being modulated. Any improvement of function is thought to stem from the adaptation of higher level processes while the newly established motor equivalent primitives remain invariant [81, 82]. However, to our knowledge, there has been no training paradigm that specifically targeted intralimb coordination following iSCI. Unloading might be a unique training pathway to target this rather specific deficit.

### Clinical relevance

Applying transparent overhead BWS to iSCI subjects during overground walking has negligible impact on CiSCI walking kinematics. This indicates that for CiSCI that retain volitional control of their limbs, but are not capable of supporting their own bodyweight, transparent BWS represents an opportunity to commence with safe, supported, unconstrained overground walking at an early time point. As patients improve, the amount of support can be reduced, and additional, perturbing forces can be applied by the therapist to maintain a challenging training environment. Furthermore, the device can be used for support while training other activities of daily life, such as stair climbing, obstacle crossing, balancing, curve walking, retrieving objects from the floor, sit to stand transitioning, etc. Overground BWS training remains only one of many rehabilitation tools, however it enables a transition from a treadmill rehabilitation environment to a more real-world setting.

### Outlook

Our conclusions are based upon a relatively small and heterogeneous sample of iSCI subjects with mainly sensory impairments due to the constraint of being able to walk 2 km/h without walking aids for approximately one hour of measurement. This walking speed was chosen as it is a viable speed for CiSCI and is close to the threshold for unsupervised indoor ambulation for CiSCI (0.6 m/s) [83]. Furthermore, at this velocity, controls still produce rhythmic, symmetrical gait, although it is far slower (~ 50%) than their typical age- and gender-specific walking velocity. A familiarization period was allowed at each BWS level and CiSCI and controls walked the target speed consistently during the experiment without transgressing the tolerance range, indicating sufficient acclimatization. While it would have been interesting to also analyze preferred walking speed or even the effects of different walking speeds, this would have led to a very long assessment protocol for the CiSCI. Baseline walking measures differed between the groups, and there was a significant age difference. Inclusion of age as a covariate in the statistical model however indicates that the adaption of gait patterns to BWS was not powered by this factor. The different responses between CiSCI and controls warrant further investigation in a wider range of individuals with CNS disorders walking at multiple speeds. Three CiSCI had TSIs of less than one year and would hence be considered closer to sub-acute than chronic. These individuals were included as they had achieved a functional plateau due to the relatively mild nature of their injuries. Functionally they ranked among the better individuals in the cohort. The investigated cohort of patients with a wide range of different impairments and TSIs provides some indication of a generalizable effect, however how this manifests specifically to level, severity and chronicity of impairment can only be determined by using more specific inclusion criteria. This limits the description of potential mechanisms in the current experiment. Going forward, we are interested in evaluating more precisely defined cohorts and better disentangling the mechanical effects from the biological responses to unloading.

## Conclusions

In synopsis, applying transparent BWS during overground walking in CiSCI from 30 to 50% bodyweight induces changes in temporal gait parameters and medio-lateral balance control, without disrupting the gait pattern or the intralimb coordination. These results are comparable to observations in controls, however controls subtly adapted their intralimb coordination in dependence on the BWS level. The predominantly sensory affected CiSCI cohort did not modulate their gait in a similar manner. Building upon these results, overground walking with transparent BWS may be a suitable extension of the locomotor therapist’s toolbox in that it can provide walking support without disrupting gait patterns in a CiSCI population. Forward-looking, investigating the adaptation to unloading may provide a window on the residual adaptive capacity of individuals with gait impairments during locomotion. While critical endpoint control parameters may behave almost normally, the underlying joint motion patterns and intralimb coordination probe deeper into the neural framework of locomotion and may provide more sensitive readouts of locomotor capacity.

## Abbreviations

10mWT: Ten-meter walk test
ACC: Angular component of coefficient of correspondence
AIS: ASIA impairment scale
AP: Antero-posterior
ASIA: American Spinal Injury Association
BWS: Body weight support
CiSCI: Individuals with (chronic) incomplete spinal cord injury
CNS: Central nervous system
CoM: Center of mass
iSCI: Incomplete spinal cord injury
LEMS: Lower extremity motor score
LT: Light touch
MEP: Motor evoked potential
ML: Medio-lateral
POS: Position sense
PP: Pin prick
RB: Romberg test
ROM: Range of motion
SD: Standard deviation
SEP: Sensory evoked potential
SSD: Square root of the sum of squared distances
TSI: Time since injury
VIB: Vibration sense
